# Remote IoT Education Laboratory for Microcontrollers Based on the STM32 Chips

**DOI:** 10.3390/s22041440

**Published:** 2022-02-13

**Authors:** Patrik Jacko, Matej Bereš, Irena Kováčová, Ján Molnár, Tibor Vince, Jozef Dziak, Branislav Fecko, Šimon Gans, Dobroslav Kováč

**Affiliations:** Department of Theoretical and Industrial Electrical Engineering, Technical University of Košice, 04200 Košice, Slovakia; matej.beres@tuke.sk (M.B.); irena.kovacova@tuke.sk (I.K.); jan.molnar@tuke.sk (J.M.); tibor.vince@tuke.sk (T.V.); jozef.dziak@tuke.sk (J.D.); branislav.fecko@tuke.sk (B.F.); simon.gans@student.tuke.sk (Š.G.); dobroslav.kovac@tuke.sk (D.K.)

**Keywords:** remote laboratory, IoT, education, microcontroller, STM32, Arm Cortex-M, remote control, remote monitoring

## Abstract

The article describes the implementation of IoT technology in the teaching of microprocessor technology. The method presented in the article combines the reality and virtualization of the microprocessor technology laboratory. A created IoT monitoring device monitors the students’ microcontroller pins and sends the data to the server to which the teacher is connected via the control application. The teacher has the opportunity to monitor the development of tasks and student code of the program, where the functionality of these tasks can be verified. Thanks to the IoT remote laboratory implementation, students’ tasks during the lesson were improved. As many as 53% (*n* = 8) of those students who could improve their results achieved an improvement of one or up to two tasks during class. Before the IoT remote laboratory application, up to 30% (*n* = 6) of students could not solve any task and only 25% (*n* = 5) solved two tasks (full number of tasks) during the class. Before implementation, 45% (*n* = 9) solved one problem. After applying the IoT remote laboratory, these numbers increased significantly and up to 50% (*n* = 10) of students solved the full number of tasks. In contrast, only 10% (*n* = 2) of students did not solve any task.

## 1. Introduction

The education sector is currently undergoing a difficult period caused by the global COVID-19 pandemic. At one point, it was necessary to completely change the form of teaching from full-time to online, which many schools and universities were not prepared for. Therefore, it was necessary to adapt teaching resources so that students and teachers had access to virtual classrooms. The most well-known online platforms are Moodle, Google Classroom, Blackboard, Microsoft Teams, Zoom, Webex, etc. Some publications state teachers’ or students’ views on the online education provided by virtual platforms, where their perception is mentioned [1,2,3]. Statistical analyses report [1] on the use of different platforms among teachers in private and public higher education institutions, where it was found that 60% of teachers had experience with virtual platforms (Google Classroom, Moodle) before teaching online and up to 80% had training on virtual platforms, which improved teachers’ ability to work with it. Another commonly used platform was Microsoft Teams; teachers and students from the Universidad del Valle de México (UVM) addressed the satisfaction of using this platform during online teaching or otherwise referred to as Emergency Remote Teaching (ERT) [2,3]. These studies proved a high reliability of and satisfaction with the tool, which is also indicated by the fact that they had no problem adapting to the use of the Microsoft Teams platform.

However, the described platforms in many cases cannot replace full-time teaching, especially when it comes to teaching medicine, dentistry, etc. Vaccination partially helped to restart full-time learning, but a virtual environment was also used [4,5,6,7,8,9,10]. In addition, a study [7] focusing on the online dental education form stated that up to 50% of respondents (*n* = 435) spent less time in education and 30% (*n* = 265) of them spent more time. Satisfaction with teaching also brought differences of opinion regarding remote learning, where 44% (*n* = 382) of respondents were satisfied and 31% (*n* = 279) of respondents were not satisfied with the current learning form.

This article focuses on microprocessor technology and electrical engineering, so the implications of a pandemic for this field will be mentioned. The study [11] states that in the field of electrical engineering, in the first months of the pandemic, communication was performed primarily via e-mail and up to 11% of exercises were not realized. Another 6% used Messenger for communication, 5% web and cloud, 4% social networks and only 2% used simulators. The study [12] states that up to 84.3% (*n* = 97) of high-school teachers were ready for the transition to online teaching, while 69% (*n* = 107) of university teachers were ready for this at the university. When asked about students’ access to classes, 57.6% of university teachers expressed concern, while only 39% of high-school teachers expressed concern about access to online teaching.

A slightly bigger problem arises in departments that require manual measurement and connection of electronic or power system solutions [13]. Courses in which the main area of focus was to teach the student to connect electronic circuits and to program microcontrollers have moved to simulation environments where they can work with development platforms such as Arduino or Raspberry Pi. In these lessons, Tinkercad online simulator is used. It is currently a widely used tool for simulating electronic circuits, similar to Proteus software, OrCad, Multisim, and other platforms. Tinkercad offers basic electronic components, sensors, platforms (Arduino, Raspberry Pi) and measuring instruments (oscilloscope, voltmeter or ammeter), with which simple circuits can be simulated [14,15]. Moreover, it also offers the possibility of the simple modeling of 3D objects. Of course, simulation presents an excellent learning method because it can be low cost and requires no physical experimental components such as electronic devices or measurement devices.

Many articles focus on experimental and simulation comparison [16,17,18], proving that experiment and simulation are consistent. The sameness is the primary requirement in the simulation, where any difference may cause fatal results. In low-cost simulators, such as microcontroller simulators, any differences have no such fatal consequences. However, it may cause a minor discrepancy between the real usage of the chips and the simulation.

The practical form of teaching is the most effective one as students can understand things correctly. Therefore, the goal of teachers is to create tools for practical teaching, whether in full-time teaching or online teaching by the virtual laboratories (VLs), which is confirmed by [19,20,21,22,23,24,25,26,27,28,29,30,31,32,33]. The publication [20] focused on mechanical engineering in India states that students can only understand the curriculum well if they experiment in the laboratory. The pandemic also moved teaching to an online form, leading to creating a virtual fluid mechanics laboratory. According to the results, the application of the laboratory has led to a high level of satisfaction on the part of both students and teachers, and the system helps to improve teaching skills. Another VL is described by Thinshe Chmunorwa et al. where the IoT education system, based on the myRIO, is used for student work to apply ambient light, proximity, and temperature sensors [23]. Jakub Svatos et al. [27] present the created Home Lab system, thanks to which students can receive practical training in electronics. In this project, the STM32 microcontroller fulfills several tasks: signal acquisition via ADC, PWM generator, voltage generator, voltmeter, and logic analyzer. The student can share the results via a remote screen and consult with the teacher. Remote learning of STM32 microcontrollers can also be mediated through the TeamViewer and STM32 Cube Monitor software, thanks to which the student can connect to and work with the microcontroller from home [28]. The hardware in the loop method, which has proved successful in the case of [29], can also be a helpful tool, where students can use the STM32 microcontroller to control power electronics circuits. This method can be beneficial in online teaching and maintain the quality of teaching even if students do not have direct access to laboratories. Due to COVID-19, and from the facts described above, it is clear that there is an urgent need to find ways to improve the quality of online learning and adapt STM32 lessons for a remote form. It is necessary to eliminate the shortcomings of remote learning and replace them with innovative tools that will improve teaching from the perspective of students and teachers. Therefore, this paper describes a unique IoT remote laboratory focused on improving microprocessor technology. The aim is to combine the advantages of simulators and real electronic components so that the quality of education is maintained even during online teaching. In the microprocessor technology course, students learn to work with the basic peripherals of STM32F446RE microcontrollers. The successful creation of an IoT remote laboratory can be an extended idea [27,28,29], where a teacher can connect to a student microcontroller during a lesson and monitor the status of his work.

Similarly, the student can work with real components throughout the lesson, which increases his knowledge in the field of programming and electronics. In addition, the IoT monitoring device can directly affect the student microcontroller by generating signals. This should increase the quality of teaching as the teacher is not only a remote observer but is a remote part of every microcontroller, which the student has in his home during online teaching. In this case, no commercial software (such as TeamViewer) is required to manage the student computer remotely.

The following chapter presents students’ answers to a survey in which they answered questions related to the online teaching of microprocessor technology. Based on these answers, a system was designed and implemented, described in Section 3. Section 4 contains the results of the implemented equipment, which was used in teaching as a prototype. Based on the device test, the results were evaluated from the point of view of students and teachers on the success of the lesson with a comparison of teaching before application and after application of the system in teaching.

## 2. Background and Motivation

**Figure 1:**
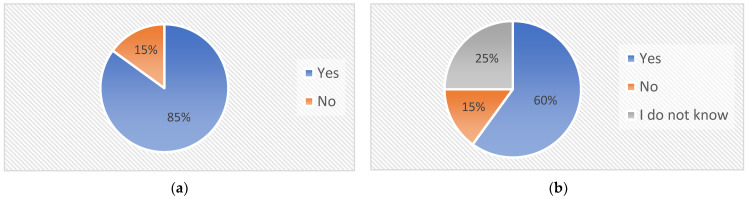
Students’ answers to questions: (**a**) “If the study was full-time, would you ask the teacher for help with programming rather than through online study?” (**b**) “Would an additional IoT module to Nucleo-64 help you, with which the teacher would monitor the status of your microcontroller, so that he could identify possible problems in solving tasks?”.

The main reason for the IoT remote laboratory creation was the problems in the online teaching of microprocessor technology. After the first weeks of online teaching, a survey was created, which was answered by students of the Microprocessor technology subject. Six questions were asked about online learning and the problems associated with this form. Twenty students answered the questionnaire with the following results:

1. Question: “*Is it difficult to study microprocessor technology online?*” 40% (*n* = 8) of students answered this question with “*yes*” and 60% (*n* = 12) answered “*no*”, which means almost half of the students had problems with online learning.
2. Question: “*Would you rather study the Microprocessor technology subject in full-time than online form?*” For this question, up to 80% (*n* = 16) answered “*yes*” and only 20% (*n* = 4) of students responded with “*no*”.
3. Question: “*Where is the biggest problem of online learning?*” Students could select more than one answer, where 50% (*n* = 10) of them responded with: “*I have trouble asking the teacher for help over the internet*” and 25% (*n* = 5) answered, “*If the teacher does not ask me how I am working, I will not say that the program does not work.*” As another problem, 30% (*n* = 6) of the students commented: “*I am stuck with the program in one place and I cannot continue*.”
4. Question: “*If the study was full-time, would you ask the teacher for help with programming rather than through online study?*” For the question, up to 85% (*n* = 17) of students responded with “*yes*” and only 15% (*n* = 3) of them selected “*no*” (see in Figure 1a).
5. Question: “*If you have a choice, would you rather learn with a simulator or real microcontroller?*” This question was important and 90% (*n* = 18) of students selected “*microcontroller*”. Only 10% (*n* = 2) selected “*simulator*”.
6. Question: “*Would an additional IoT module to Nucleo-64 help you, with which the teacher would monitor the status of your microcontroller, so that he could identify possible problems in solving tasks?”* This was the most important question. Up to 60% (*n* = 12) of students would welcome additional hardware for microprocessor technology learning. A total of 15% (*n* = 3) of students answered “*no*” and 25% (*n* = 5) responded with “*I do not know*” (see in Figure 1b).

Based on the answers obtained, it was clear that it was necessary to adjust the subject so that the teacher would be in better contact with students throughout the lesson.

## 3. Materials and Methods

The designed remote IoT laboratory consists of hardware parts and software parts. It can be stated that only a simulator or virtual laboratory is not enough as it is necessary to teach hardware connection and software programming. The primary parts of the system are the STM32 microcontroller with a high-speed Arm Cortex-M processor, a single IoT module named esp8266 used for internet communication, and an application for the teacher focused on monitoring and controlling the student’s microcontroller implemented on the Nucleo-64 board. The system is primarily focused on Nucleo-64 development kits. For the idea, functions on the remote IoT system help a teacher with teaching, where he will have complete control over the student microcontroller. This makes it possible to control and watch the student activity during the lesson.

The IoT monitoring device sends information about the student microcontroller program that the student is working on. The system also includes a function for examinations such as finding the voltage generated by the teacher control application through the IoT monitoring device or finding the period and duty cycle of the PWM signal. These factors are novel as they connect simulation and real experiment, which is excellent for remote education.

### 3.1. Hardware Parts

As mentioned, the system’s primary parts include the STM32 microcontrollers and esp8266 module used for communication with a server, which creates a network connection between the teacher and the students.

**Figure 2:**
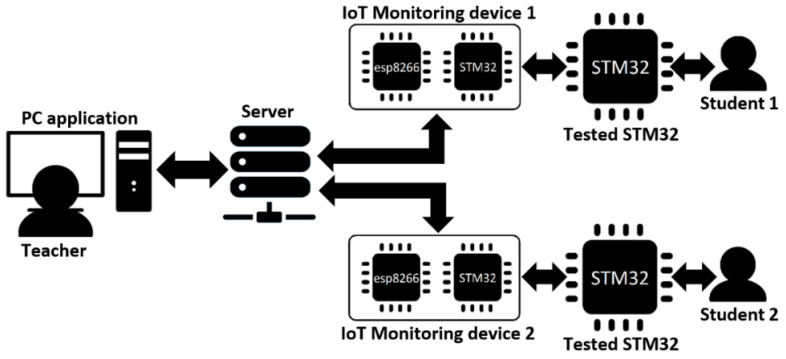
The IoT remote laboratory design in blocks.

The STM32F446RE microcontroller the students used for learning microcontroller programming is the high-performance chip of the STMicrocontroller company focused on microelectronics components. It includes an Arm Cortex-M4 core specially designed for digital signal processing. The microcontroller was selected for the education program due to performance, DSP instruction set, and multi-ADCs functions, which makes it possible to speed up the AD conversion by several times [34]. Per Figure 2, two STM32F446RE microcontrollers create a student hardware part, where the first STM32F446RE microcontroller performs monitoring functions and the second is the same microcontroller students use for its tasks.

The esp8266 chip implemented on the ESP8266MOD module of the Espressif system company is used for internet communication performance. It is necessary for each IoT monitoring module to connect the control application to the student module. The esp8266 is one of the simplest esp chip/module family modules, which operates on 32-bit RISC Tensilica L106. Its 2.4 GHz ratio consists of receiver and transmitter high-speed clock generator and crystal oscillator. The primary advantage of the chip is TCP/IP protocol implementation with 802.11 b/g/n WLAN MAC [35]. Because the esp8266 performs communication via AT commands, only RX, TX, VDD and GND, pins must be connected with the scanning STM32F446RE microcontroller.

**Figure 3:**
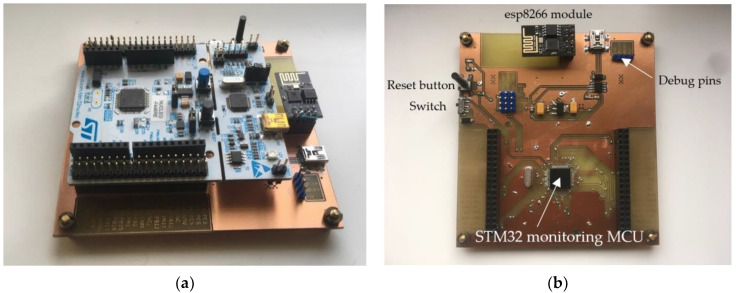
A hardware part of the IoT remote laboratory: Student Nucleo-64 board connected to the IoT monitoring device (**a**) and created PCB (**b**) with equipped monitoring microcontroller STM32F446RE, IoT module esp8266, and the other required components.

As it is possible to see in Figure 3a, the upper Nucleo-64 board with STM32F446RE microcontroller (student microcontroller) is retracted to the IoT scanning module. A PC powers the Nucleo-64 board through a USB connector used for student PC—microcontroller communication, and the communication is provided by the USART peripheral. The student Nucleo-64 board powers the IoT monitoring device via its power supply pins. After connecting the IoT monitoring device and Nucleo-64 board and turning on the switch, the scanning module is powered and ready for operation. Figure 3b shows the created PCB of the IoT monitoring device with the STM32F446RE microcontroller, esp8266 module, reset button and the turn-on switch. The PCB also includes crystal, debug pins and a USB connector for PC connection possibilities. SWDIO, SWCLK, and GND pins are used for firmware updating, shown in the upper right corner.

All the I/O pins of the monitoring STM32 microcontroller have the same position as the student Nucleo-64 board without PA9 and PA10 pins. These pins are specially used for USART1 communication, where PA9 as RX pin of the monitoring module is connected to the PA10 of student’s Nucleo-64 board. The same change is performed at the PA10 as the TX pin of the monitoring module to the PA9 as the RX pin of the Nucleo-64 board.

### 3.2. Software Parts

The software part of the remote IoT laboratory consists of three programs. The first is the monitoring microcontroller program connected to the student microcontroller implemented on the Nucleo-64 board. The second is the server program used for data collection from the teacher control application and student IoT monitoring device. This program, installed on the server, is presented as a link between teacher and students. All the information about the students’ programs is stored in it. The server program is required because a public static IP address is necessary. Nowadays, all households and offices use a private internet network, where all PCs have privately dynamic IP addresses, which is usually 192.168.xxx.xxx (if C class is used). Of course, the server would not be required if all the students and teachers would be in the same classroom. In this case, the teacher application would be a server and all the microcontrollers would be connected to it. However, the primary aim of the remote IoT laboratory is regarding remote education, where a server with a public IP is required.

#### 3.2.1. Server Program

Because the server and its program work as a primary linker between teacher and students, this part contains detailed information about the server program.

The server program, programmed by Microsoft Visual Studio 2019, fulfills several functions:

- Starts the server;
- Connects the teacher application (control application);
- Connects the student IoT monitoring device;
- Collects stored information about student connections during the lesson;

After the server starts, the program waits for clients to connect. In this case, the control application and IoT monitoring devices represent the clients. For client recognition, both the control application and IoT monitoring device send a unique command with its IP address. After the successful connection of the control application, the application sends a “teacher” command, while the IoT monitoring devices send “studentx” commands, where “x” represents a unique number of the module. The server program then stores the IP addresses into the array (IP_address_array) to the position corresponding with the IoT monitoring device number. The control application’s IP address is stored in the zero position of the array. The IoT monitoring device’s IP address, referred to as “student1”, is stored in the first position, “student2” to the second position, etc. The module number is important for the teacher because he may not know the IP address of the student. However, it is enough for him to know the IoT monitoring device number assigned to the student he wants to communicate with. When a student is connected to the server, the server program saves the connection time to the report.txt file. The report stores all the connections and disconnections of each student’s IoT monitoring devices. The teacher is then informed about the students’ participation in the lessons and can thus check the entire semester.

The second report file is “task_report.txt”, which stores successful or unsuccessful information about the tasks that students had to complete during the lessons. The teacher can thus monitor the success of the assigned tasks during the semester.

Without a storage function, the server program operates as a linker between teacher and students. Therefore, forwarding commands and responses is one of its primary functions.

Depending on the initial character of the command, the server decides to whom the message or command is addressed. A teacher does not need to know the IP addresses of the students. Therefore, he needs only to know the numbers assigned by the IoT monitoring device. Then, the server connects the unique number of the module with its IP address and sends it a command from the control application. The server also proceeds to send a response from the IoT monitoring device addressed to the control application. Addressing commands or responses to a specific IP address, whether leading characters secure the control application or the scanning module:

- Control application to student IoT monitoring device: “sx-y”, where “*s*” represents addressing a student with an IoT monitoring device number corresponding to “x”. The “y” character represents other data which are transmitted.
- Student IoT monitoring device to control application: “tx-y”, where the recipient is a teacher by the first “t” character. The “x” indicates the IoT monitoring device number from which the response comes from.

**Figure 4:**
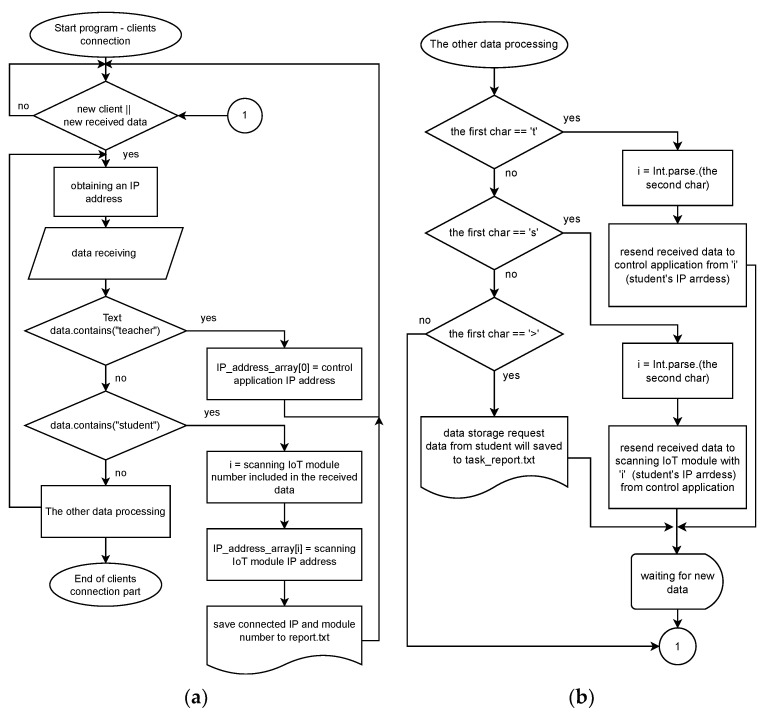
The shown flowchart describes the server program operations from the start of the server up to received data processing. The first flowchart (**a**) describes how clients connect to the server and save the data to a text file. The second flowchart marked as (**b**) is part of the first flowchart where the block “The other data processing” is extended and described.

The flowchart shown in Figure 4 shows that the server program waits for a new client connection or the incoming data. The first character or sequence of characters represents its type of data command. Depending on this character, the server recognizes where the message should be addressed. Of course, if the server program receives the first character “>”, it means that all the following characters representing the data should be saved to a report file. These data represent the results of a student program that emerged during a lesson or exam.

#### 3.2.2. Control Application

The control application used by the teacher presents the primary control part of the whole device. The application includes several control/test parts, such as I/O pins or peripherals testing. The school subject, where the IoT system is used, includes GPIO (General-Purpose Input Output) learning, timers where PWM is generated, or Input Capture mode is applied. In this mode, students are tasked to determine the length of time between two events or to measure the period and duty cycle of the PWM signal. The A/D or D/A converters are the other essential peripherals that the system includes. Moreover, the last part is the USART/UART communication peripheral between the control application and the student microcontroller.

The control application was created by Microsoft Visual Studio 2019, such as a server program.

**Figure 5:**
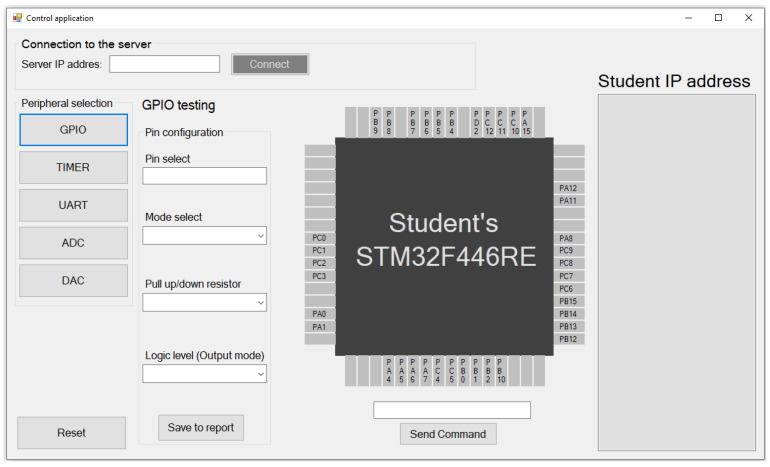
The control application in its start screen—GPIO testing.

In Figure 5, the teacher control application is shown, which consists of four primary parts:

- *Connection to the server*, shown in the left upper corner, where the user (teacher) enters the server IP address. In this case, two parameters are required—server IP address and port. Both parameters are static. Therefore, it will not be changed (unless the application is on another server with a different IP address). After the connection, the program informs the user about a successful connection;
- *Student IP address* is used for the students’ IP address and module number display connected to the virtual laboratory. This list is refreshed each time a new student joins. The teacher sees the number of the IoT monitoring device and the corresponding IP address of this module. By clicking on a specific module number, the teacher selects the module with which he wants to communicate;
- *Peripheral selection* is used for peripheral selection, where the teacher can choose GPIO, TIMER, USART, ADC, or DAC peripheral. By clicking on a button, specific peripheral testing is shown. In this case, it is GPIO testing;
- *The peripheral testing* shown in the middle of the application has specific parameters. While the GPIO shows all the usable pins of the microcontroller with pin configuration such as pin mode, pull up/down resistor activation, etc., TIMER peripheral window includes period configuration, duty-cycle, timer mode which means Input capture or Output Compare (PWM). Using a timer, of course, requires a timer number and channel selection. The UART testing includes a text box for data receiving in string form and a transmit text box for characters or strings that the teacher wants to send to the student microcontroller. Of course, ADC and DAC testing include parts with measured voltage blocks or voltage values that the teacher wants to generate by IoT monitoring device for students. All peripherals can work together. The other peripheral testing windows will be shown in the Results and Discussion chapter.

**Figure 6:**
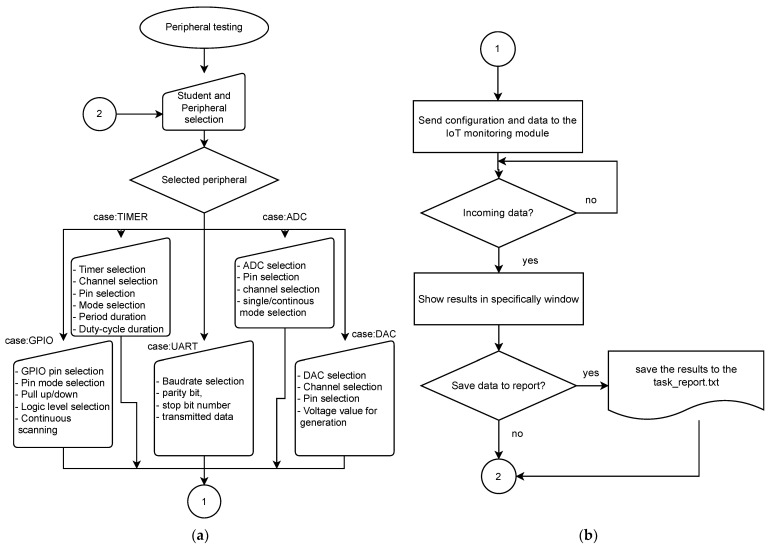
The shown flowcharts mainly create control application functions. The first part marked as (**a**) describes the selection of the tested peripherals and the manual input of peripheral configurations, or the selection of data sent to the server and subsequently to the IoT monitoring device. The (**b**) is a continuation of the first part of the algorithm, which describes the method of receiving the response from the IoT monitoring device, its display and storage in the report.

The flowchart shown in Figure 6 describes the primary function of the application. It is the part in which the teacher selects the IoT monitoring device of a particular student and selects the peripheral he wants to test/control. In the case of testing, he can choose a specific IoT monitoring device pin that is the same as the student use and find out its logical value. Also, during testing, he can find out if the student has configured the PWM signal correctly, for example, on the PA1 pin. The teacher sets the same IoT monitoring device pin to capture the period and duty cycle to capture the PWM signal generated by the student microcontroller. The input capture mode of the TIMER peripheral is used for such monitoring. The device can also be used for control, in which the IoT monitoring device sets specific parameters that the student must measure. For example, the teacher will generate a voltage of 1 V using a DAC converter on a PA0 pin. In this case, the student has the task of using his microcontroller on the PA0 pin via the ADC to determine what voltage was generated by the IoT monitoring device. The student will only find out this voltage if his microcontroller is configured correctly. This method of management is very suitable for student exams.

The following table (Table 1.) shows the procedure for connecting and sending the configuration settings and data correctly.

#### 3.2.3. IoT Monitoring Device Program

**Figure 7:**
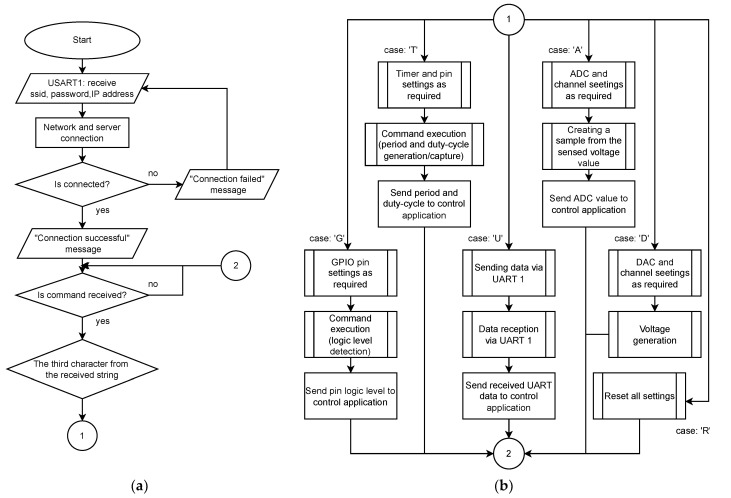
The last flowchart shows how the IoT monitoring device works. The flowchart consists of two parts, where (**a**) is the first part and (**b**) is the continuation or the second part.

The IoT monitoring device program is the last software part. Its primary task is to wait for control application commands and execute them. If any command was received, the program executes the request of the control application by configuring the selected peripheral according to the received parameters, scans the required pins and immediately sends the result to the server, which then forwards the message to the control application. Before the mentioned operations, it is necessary to connect the device to the network and the server. The student Nucleo-64 board provides network and server connection through the STM32 microcontroller. The USART2 and USART1 peripherals are used for this. Through the USART2 peripheral, the student microcontroller communicates with the PC and the terminal. All data containing the network settings and the server connection must be forwarded to the USART1 peripheral, connected to the same peripheral of the IoT monitoring device. The connection to the network and the server is made via a command: +ssid;password;ip%. The student microcontroller receives the command sent from the student PC. This command is sent to the IoT monitoring module, which uses SSID and password to connect to the network. The module then connects to the server via the IP address and port (fixed and stored in the IoT monitoring device memory). The IoT monitoring device informs the student about the success/failure of the connection via a message sent to the student microcontroller. After the connection, the IoT monitoring device is ready for use. The whole functionality of the IoT monitoring module is described by the flowchart in Figure 7.

Monitoring the STM32 microcontroller makes use of a USART2 NVIC (Nested Vector Interrupt Control) for immediate command processing. This means that if any data are received, the microcontroller will process it immediately thanks to interrupts. After the command processing and the peripheral and pin configuration, the microcontroller provides the required operations. Results are transmitted to the server, which forwards them to control the application with a particular IoT monitoring device number. The response from the IoT monitoring device is in the illustrative format: “t4A3500%”, where “t” represents the recipient (teacher in this case) and number “4” is the IoT device number. “A” represents the ADC peripheral that created the sample that indicates that a value is 3500. This number represents the data part of the response. A “%” is the last character of the response.

**Figure 8:**
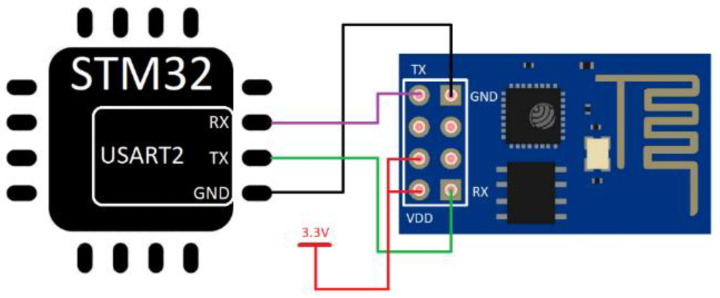
Monitoring STM32 microcontroller and esp8266 module connection via USART2.

All other modules work the same way. The critical role of the project is the esp8266 module, which provides internet communication and connection with the server. Without this module, creating a remote laboratory would not be possible. Connection between monitoring microcontroller and esp8266 is shown in Figure 8.

The IoT scanning device includes two essential parts—monitoring STM32 microcontroller and esp8266 module, which communicate via AT commands, where UART communication is used. Therefore, all data, commands, or responses are transmitted between those parts. The esp8266 works as a linker that repeats the messages. The USART2 peripheral of the monitoring microcontroller is used for communication.

### 3.3. IoT Remote Laboratory Applaing to the Education

The primary reason for creating and applying an IoT remote laboratory to education was mentioned in the survey, where students said that a monitoring device can solve their programming problems. An IoT remote laboratory was applied for testing in education between the 9th to 12th week of the semester. AD converters, DA converters, PWM signal generation, and microcontroller application in practice were learned over these weeks. Each student received the Nucleo-64 board, IoT monitoring device, and electronics components for home learning, where they followed the connection according to Table 2. During the nineth week of the semester, when the IoT remote laboratory was first applied, connection problems occurred as the template with the STM32 code was missing and students had to manually insert USART peripheral codes for connecting to server. This problem was solved the next week through the template STM32 code for each student. The template includes a clock configuration, and USART1 and USART2 configurations. USART2 was added between PC and student microcontroller communication and USART1 provides student microcontroller and IoT monitoring module communication. Thanks to the STM32 template code, connection problems were solved and students could easily connect through the “Termit” terminal software in which they entered the connection command. Another problem that occurred during the first use of the IoT remote laboratory was the reset command being missing for IoT monitoring device pins reset. In this case, a system reset was required via a manual reset button. This problem was repaired by an IoT monitoring device firmware update and by inserting a reset button into the control application.

The lessons took place using Webex software, where the teacher was in audiovisual contact with the students. After starting the lesson (by connecting to Webex), the students connected the Nucleo-64 to the IoT monitoring device according to Table 2. When all participating students were connected to the IoT remote laboratory server, they were given two tasks to work out during the lesson. While working on the tasks, the teacher monitored the progress of the students’ work via the IoT monitoring device. If it was clear that a student had not progressed on the task for a long time, the teacher immediately contacted him and began to solve his problem with the task and the code. This resolves part of the survey, where students stated that “If the teacher does not ask me how I am working, I will not say that the program does not work”. Other problems were also resolved, where the students replied stating that they had a problem asking the teacher for help via the Internet or with “I am stuck with the program in one place and I cannot continue”. In this case, the created system proved to be effective and helped to solve problems with the tasks; the students were approached and were able to work on their problem. In this way, the number of solved tasks during the lessons increased (the results are given in the Results and Discussion chapter).

## 4. Results and Discussion

The functionality of the IoT remote laboratory was tested using one IoT monitoring device, by the control application and a server to which both parts connect. Testing took place in the laboratory, with the server and server application running a remote site accessed via a remote desktop.

The first step of testing was to connect both sides to the server. The teacher computer was connected via a control application and the student side was connected via a terminal.

**Figure 9:**
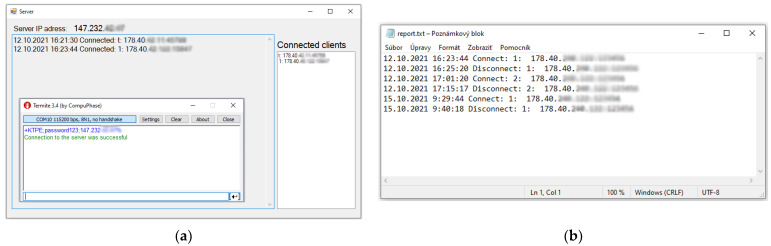
(**a**) The application on the server displays the connected devices. The Termite application (terminal) indicates the success of the IoT monitoring device connection. (**b**) Connection report from the server.

Figure 9a shows the server application on which the connected devices are visible in the “Connected clients” section. This window shows the IP address of the teacher marked “t:” and the student marked “1”, which represents module number 1. The application main window displays the report of connection and disconnection from the server. After a successful connection, the student can work on his tasks and the teacher can follow the development of the program on which the student is working by testing the peripherals. Of course, it depends on the currently covered topic, according to which the teacher chooses the peripheral he wants to monitor. In Figure 9b, all the stored connections and disconnections are shown in a report.txt file.

### 4.1. Functionality Results

**Figure 10:**
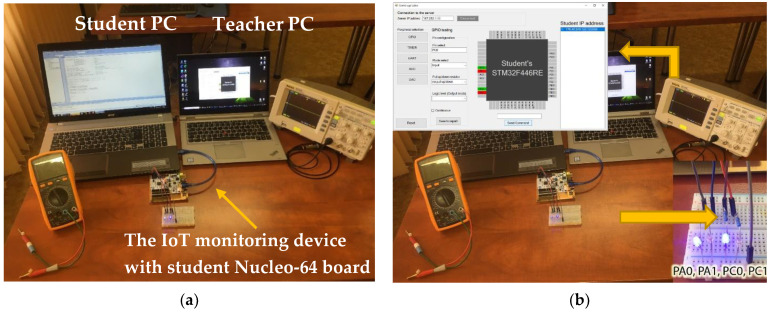
(**a**) Workplace of a remote laboratory with a teacher PC, a student computer, and the IoT monitoring device with measuring devices (multimeter and oscilloscope). (**b**) GPIO pins monitoring where PA0, PA1, PC0, and PC1 pin was tested.

The experiment involved testing all the proposed peripherals. In addition to the basic concept of the modules and devices used, measuring instruments such as the Rigol DS1052E oscilloscope, the AXIO AX-582B multimeter, and high-brightness LEDs were also used. The workplace is shown in Figure 10a, where the student Nucleo-64 developing board is connected to the IoT monitoring device and the student PC. The server connects the control application of the teacher PC and IoT monitoring device.

The second part of the results compares teaching and tasks completed before and after the application of IoT remote laboratory. This part contains the comparison describing all the findings and system advantages.

GPIO was tested as the first peripheral, where it was necessary to find out the logical level of PA0, PA1, PC0, and PC1 pins.

The following Figure 10b shows GPIO peripheral testing. As can be seen, the student microcontroller program has been programmed to have a high logic level on PA0 and PC0 pins and a low logic level on PA1 and PC1 pins. The same result was captured on the control application, where HIGH logic pins are drawn in green and LOW logic pins are shown in red. Because the student set the pins as OUTPUT, the teacher had to set the same pins as INPUT on the monitoring device to read the logic level of the student pins. 

**Figure 11:**
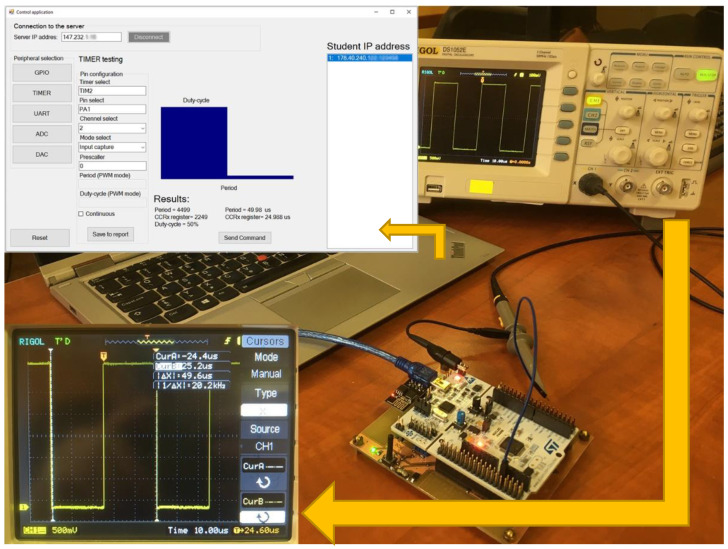
PWM monitoring provided by timer TIM2. The oscilloscope captures the signal showing 50 µs periods with 50% duty-cycle. The results are shown in the upper left corner of the control application.

The second test was performed on the PWM signals, where the timer peripheral was tested. In this test, the student microcontroller generated a PWM signal on the PA1 pin, with a 50 µs period and 50% duty-cycle. The generated PWM signal was monitored by a 32-bit and 90 MHz timer of the IoT monitoring device. In the experiment, the Input capture mode of the timer was selected. This mode is designed to capture the signal rising and falling edges that the PWM signal contains (see in Figure 11). 

The control application shows two results: captured counts period, equal to 4499 and duty-cycle in the counts equal to 2249. As mentioned within the lesson, it is possible to task a student to find the period and duty-cycle of the PWM signal (with Output compare mode selected) generated from the IoT monitoring device.

The timer peripheral and Input capture mode can also measure time base or event timing (for example, blinking LED frequency).

**Figure 12:**
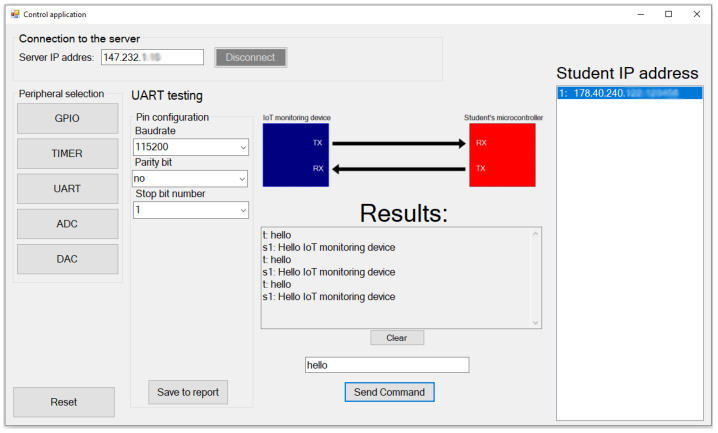
UART peripheral testing. Communication took place on baud rate 115200 without parity bit and one STOP bit.

The UART peripheral was tested via USART1 of both microcontrollers. The student microcontroller is programmed, so that if a “hello” string is received, the student microcontroller will respond: “Hello IoT monitoring device”. This message is transmitted to the server and forwarded to the control application. The result is shown in Figure 12.

The UART peripheral test is possible only via USART1 shared on the PA9 and PA10 pins. The teacher can thus send any command to the student microcontroller, on which the microcontroller will perform the required operations.

The following test was performed on the ADC peripheral. In this case, the IoT monitoring device was set to ADC mode. ADC1 and channel 4 were selected on the PA4 pin. A voltage of 2.01 V was generated on the same pine from the student microcontroller via the DAC.

**Figure 13:**
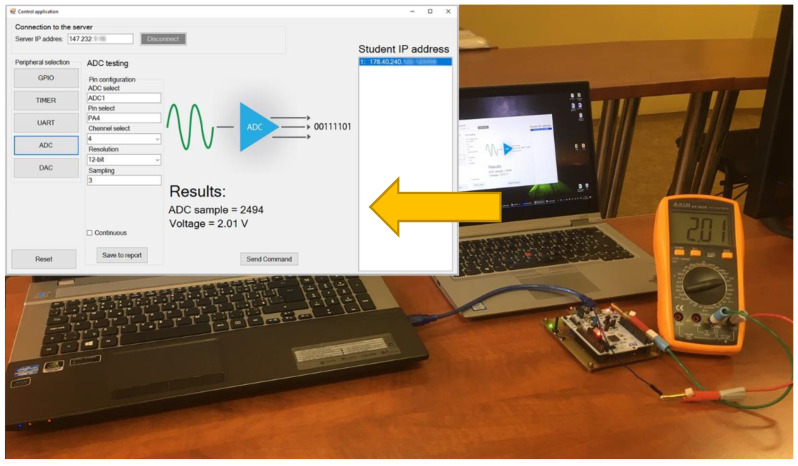
ADC peripheral testing.

As shown in Figure 13, the IoT monitoring device measured the same voltage as shown on the voltmeter. The sample, which was captured by the IoT monitoring device, is displayed in digital form and the voltage, which the application automatically calculates from is in the digital form.

A 12-bit resolution was used with 3ADCCLK sampling and a reference of 3.3 V in the experiment. It is a necessary to note that 30 MHz is the ADC operation frequency. The ADC testing allows to teach single and continuous modes. In the single-mode, only one sample is created. In the continuous mode, the ADC makes the samples cyclically and sends them to the control application.

The DA converter was the last tested peripheral. In the experiment, the teacher, via the IoT monitoring device, generates voltage (within the reference voltage range, which is 3.3 V). The student’s task is to detect the voltage through ADC at the same pin, which the student can only find if he sets up his ADC correctly.

**Figure 14:**
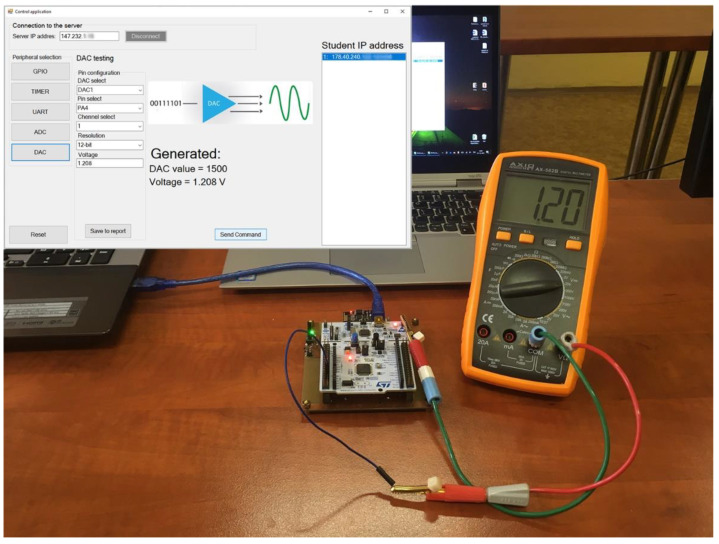
DAC testing with 12-bit DAC1, channel 1 generated through PA4 pin.

The Figure 14 shows DAC testing, where voltage is generated by the IoT monitoring device and measured on the student’s Nucleo-64 board. In this test, the voltage was set to 1.208 V, equal to 1500 for a 12-bit DAC. The voltmeter in the figure shows the voltage on the student microcontroller pin. The voltage is identical to the required voltage generated by the IoT monitoring device. The student task would be to measure this voltage with the AD converter.

The DAC peripheral configuration consists of the DAC selection, channel and pin selection. In this case, only PA4 for channel 1 and PA5 for channel 2 is possible. It is possible to see that channel 1 and PA4 pin were selected. The “Voltage” textbox in the control application includes the required voltage, which the IoT monitoring device will generate.

The most robust feature of the IoT remote laboratory is the student’s ability to practically connect the electronics circuits and program a microcontroller from home during the online lessons. The student does not lose contact with real projects, as often happens in the context of employing simulators. Therefore, the IoT remote laboratory has a much more significant benefit for the student than the TinkerCad simulator [14,15]. Moreover, the mentioned simulator does not contain the possibility of programming STM32 microcontrollers, which is focused on microprocessor technology. Among the projects similar to the described IoT remote laboratory are mainly [27,28,29]. Compared to the remote teaching system described in [27], the IoT remote laboratory has the advantage of the teacher being able to monitor any student microcontroller at any time. A teacher selects the student’s IoT monitoring device number and checks the functionality of the student’s microcontroller. The project described in [28] has a good idea, where one microcontroller is used, to which students can connect via the TeamViewer software. The advantage of the IoT remote laboratory over this system is that students have Nucleo-64 and electronic components at home and can work independently, connect circuits, and program their microcontrollers without connecting to a shared remote microcontroller. The hardware in the loop described in [29] represents an excellent idea for online teaching, where students do not have access to the laboratory. However, thanks to the described device, they can work with power electronics via STM32 at home. The disadvantage of this system compared to the IoT remote laboratory is that the teacher does not have the possibility of a remote connection to the system and cannot help the student better. The main disadvantage of the IoT remote laboratory is the problem of having access to the student code of the microcontroller, which is currently solved by sharing a student desktop. This problem may be solved in future work.

### 4.2. Educational Results

The results obtained by the IoT remote laboratory were very pleasing. Thanks to monitoring student tasks, many stagnations were found. Therefore, it was clear that the student had a problem with the task for which he was immediately addressed and his problem was subsequently solved. Such monitoring succeeded in achieving a much larger number of solved tasks than before the application of the IoT remote laboratory.

The following table (Table 3) contains data about the average success of solving tasks, where 20 students participated in the lessons. They had to solve two tasks per lesson.

The table provides an average overview of the success of solving tasks during the lesson. As can be seen, online teaching is a sign of the success of solved tasks, where up to 6 students out of 20 did not complete any task, and up to nine students completed at least one task. This was most affected by the problem of getting stuck in a piece of code or a problem for which the student required help (as mentioned in the survey). It was also difficult for the teacher to tell if the student was bothered with the task, did not know how to start, or had only a minor code problem. With the IoT remote laboratory application, the teacher had much more significant possibilities with monitoring the progress of the tasks. If it was found that the essential functions of the task still did not work, the teacher contacted the student via Webex, who told him about his problem with programming. He then shared a screen with the code of the task at hand. After finding a mistake or instructions, the student continued his work, which described the number of successfully solved tasks. Up to 10 students were able to solve the full number of tasks, eight students solved one task, and on average, only two students did not solve any tasks.

In addition to monitoring student microcontrollers, a control task via the IoT monitoring device was created. The students’ task was to determine the value of the period and the duty cycle of the PWM signal through the Input capture mode of the timer TIM5. Each student IoT monitoring device generated a 25 kHz PWM signal on the PA1 pin with a 30% duty cycle. This task aimed to determine the usability of the PWM signal generation mode. It was found that eight students did not need any help in determining the period and duty cycle. The other students needed help, with up to nine students finally completing the task and determining the required parameters. Despite help, three students did not complete the task. The results of both tests are shown in Table 4.

The results were better regarding the determination of the voltage generated from the IoT monitoring device, where the students had to determine the voltage value on the PA5 pin via the ADC. The DAC of IoT monitoring device generated a voltage of 1.75 V, and only one student did not solve this task. Eight students did not need help in this case, and 11 needed simple help. It was found that the most common error was inattention, where students with up to 60% error rate (among other errors) forgot to call the peripheral configuration to the main() function.

### 4.3. Discussion

The most significant benefit of the IoT virtual laboratory is the possibility of higher efficiency of remote education, caused by the COVID-19 pandemic. Of course, it is possible to transfer microcontroller programming learning to an entirely virtual environment through simulators, but the aim of this project was to avoid them. Up to 90% of students (n = 18) were for this form of teaching from the mentioned survey, while only 10% (*n* = 2) were for the simulator. The most significant benefit of the lesson (microcontroller technology) is the physical circuits connected to the microcontroller and the real testing, measurement, and creation of simple projects.

**Figure 15:**
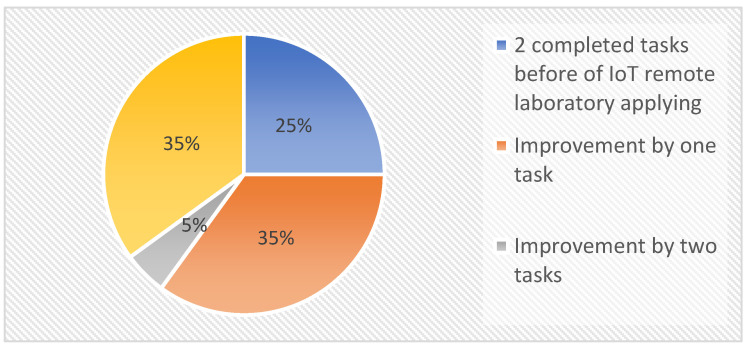
Graph of task improvement after IoT remote laboratory application.

The success from introducing the device into teaching is also confirmed by the graph shown below (Figure 15), which shows the number of students and their improvement in solving the task during teaching.

Based on the results obtained from recording the success of the solved tasks, an analysis was created, which discusses in detail the improvements of individual students.

**Figure 16:**
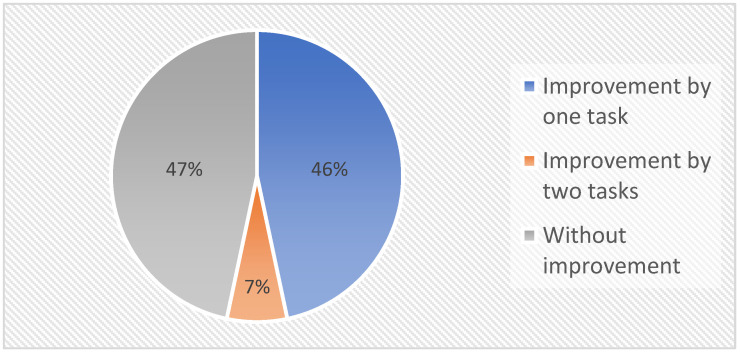
Students who were able to improve their performance in completing tasks.

The best students were able to solve two tasks regardless of whether IoT remote labs were implemented in teaching or not. On average, there were five students, which increased by another five students following the application of the IoT remote laboratory. Most of the newly added students (four students) who solved two tasks were from the group “one solved task”, and one student who did not solve any task before the IoT remote laboratory application. In these cases, mostly minor mistakes hindered the students from better results. However, before applying IoT remote laboratory, it was not easy to observe and correct these errors. The group in which the students solved one task “before implementation” was left with five students who did not improve their results, and three students from the group “zero completed tasks” improved their results. These students usually had more serious problems with peripherals configuration or with the functionality of the task, with which they had been struggling for a long time. On the positive side, however, the group with “zero completed tasks” shrunk significantly. These students had great difficulty understanding the new curriculum, which was reflected in their results. The results of the students’ improvement are shown in Figure 16.

It was also known from the results recorded for individual students that in most cases, the improvement still occurred in the same students, as well as in the same students who had stagnated tasks and did not achieve improvement. Therefore, it was relatively easy to result in average improvements per 4 weeks of lessons with the IoT remote laboratory.

## 5. Conclusions and Future Work

This article dealt with designing, implementing, and testing a virtual IoT laboratory designed for the remote education of microcontroller techniques. The laboratory was designed so that students still had access to real electronics components, microcontrollers, and measuring devices and so that they did not have to transition to fully simulated teaching. The student and the teacher could connect to the server through the created server and its application, thus mediating the teacher-student connection. Through the IoT monitoring device, the teacher could monitor students’ work during the lesson or enter tasks that the student has to solve. The IoT monitoring device stored each student login and logout into the report, allowing the teacher to monitor class activity during the semester.

The main advantage of the device is the simple implementation by the student, who plugs his Nucleo-64 into the IoT monitoring device, turns it on, and connects to the network. Subsequently, he can work as he usually does during the full-time form of teaching. On the contrary, the teacher gains many benefits that will help him understand the student’s perspective.

From the above findings, it can be concluded that up to 40% (*n* = 8) of students of the group increased in their success of solving tasks during the class. In comparison, 25% (*n* = 5) of the total group of students performed two tasks before the implementation of the IoT remote laboratory, and 35% of the total students did not improve their performance. As a result, a total of 15 students were able to improve, of which up to 53% (*n* = 8 of 15 students) had improved results.

The results thus show a decent increase in successfully completed tasks, which IoT remote laboratory is responsible for. The proposed system can be extended and implement additional functions or extend the IoT monitoring device with other types of microcontrollers and development boards, which could further increase the success of the task during the lesson. In general, however, all types of Nucleo-64 boards with the same pinout (as NUCLEO-F446RE) can be connected to the device. The system can be extended by implementing Nucleo-32 development boards inserted into the IoT monitoring device via a unique bridge or driver. It would also be possible to monitor the esp32 or Arduino platform, where there would have to be a driver for a different voltage logic. PLC devices could also be connected to the system, monitored and controlled through a voltage driver. The device can also be expanded with additional peripherals. The addition of other peripherals can include SPI, I2C, CAN bus, and the addition of other modes already included in the peripherals such as triangular voltage generation and other shapes in the case of DAC, etc. As mentioned in Section 4, another major extension of the project may be the teacher’s direct access to the student’s microcontroller code via loading and sending a main.c file.

The described system can thus be fully applicable to teaching practice in the future, whether in remote or full-time education. Therefore, we believe that the IoT monitoring device will find application in a broader focus of microcontroller education and will help in teaching, and subsequently increasing the number of successfully completed tasks.

## Figures and Tables

**Table 1:** Procedure for using the control application—teacher’s part.

Step	Action
1	Start the program
2	Enter the IP address of the server
3	Click the “Connect” button and wait for the connection
4	Select the IoT monitoring module number from the “Student IP address” block
5	Select the peripheral, pin, and all required configurations
6	Press the “Send command” button
7	Wait for received data from the IoT monitoring device
8	If it is required, save data to the report or reset all current configurations via a particular button

**Table 2:** Procedure for using the IoT monitoring device—student’s part.

Step	Action
1	Connect student Nucleo-64 to the IoT monitoring device
2	Connect Nucleo-64 board to the PC
3	Turn on the IoT monitoring device using the switch
4	Send a command to connect the module to the network
5	If the connection is successful, the student can work on their tasks

**Table 3:** The average success of solving problems before and after the application of the IoT remote laboratory.

Number of Completed Tasks	Student Number before IoT Remote Laboratory Applying	Student Number after IoT Remote Laboratory Applying
2x	5	10
1x	9	8
0x	6	2

**Table 4:** Solving the task of detecting PWM signal and voltage from IoT monitoring device.

Task Completion	PWM and Duty-Cycle Detection	Voltage Detection
Without help	8	8
Resolved via IoT remote laboratory	9	11
Unresolved	3	1

## Data Availability

The data presented in this study are available on request from the corresponding author. The data are not publicly available due to study continues and the data is still being worked on.
